# Dataset concerning haematological and biochemical parameters changes in show jumping horses subjected to exercise and plasmapheresis session

**DOI:** 10.1016/j.dib.2019.104653

**Published:** 2019-10-12

**Authors:** Réda Daden, Sara Chbihi, Fatima Zahraa Zarhouni, Jamal Chakir, Mohammed Piro, Mohamed Rachid Achaaban, Mohammed Ouassat, Khalid El Allali

**Affiliations:** a Comparative Anatomy Unit/URAC49, Department of Biological and Pharmaceutical Veterinary Sciences, Hassan II Agronomy and Veterinary Medicine Institute, Rabat, Morocco; b Jumenterie de Zouada, Veterinary Hospital - Royal Guard, Tetouan, Morocco; c Veterinary Hospital- Royal Guard, Rabat, Morocco; d PMC- EC, Department of Medicine, Surgery and Reproduction, Hassan II Agronomy and Veterinary Medicine Institute, Rabat, Morocco

**Keywords:** Plasmapheresis, Horse, Exercise, Haemato-biochemical parameters

## Abstract

This article presents data on the effect of plasmapheresis on clinical, haematological and biochemical parameters in horses following exercise and after a plasmapheresis session. This blood filtration technique was realised on six jumping horses (plasmapheresis group) that underwent three consecutive days of graded physical exercise. The control group (n = 6) went through the same exercise but was not subjected to the plasmapheresis session. Blood was sampled before and after each exercise, also at the beginning and the end of plasmapheresis session. The presented data was obtained by measuring clinical and haemato-biochemical parameters in both groups. The heart and respiratory rates and rectal temperature were recorded. In addition, the number of red blood and white cells, platelets also of lymphocytes, monocytes, eosinophils and granulocytes were counted. Other haematological parameters including, hemoglobin concentration, hematocrit, mean corpuscular volume, mean corpuscular hemoglobin, mean corpuscular hemoglobin concentration were determined. Concerning the biochemical parameters, the concentrations of albumin, globulin, total protein, glucose, alkaline phosphatase, aspartate aminotransferase, gamma glutamyl transferase, total bilirubin, lactate, creatinine kinase, urea, creatinine, calcium, sodium and potassium were measured. All parameters data were analyzed by a two-way repeated measures analysis of variance followed by Holm-Sidak post-hoc procedure to evaluate the effect of plasmapheresis and time. This paper contains data related to and supporting research articles currently published entitled “Plasmapheresis effect on haematological and biochemical parameters in athletic horses subjected to exercise” (Daden et al., 2019) [1].

**Table:** Specifications Table

Subject	Veterinary Science and Veterinary Medicine
Specific subject area	Equine medicine, Equine exercise, Exercise physiology, Blood filtration technique, Plasmapheresis, Haematology Biochemistry
Type of data	Tables, Figure
How data were acquired	-Physiological parameters: Physiological parameters were measured in the two groups using the same procedures as in clinical examination. • Heart rate: the pulse on the facial artery was taken. • Respiratory rate: Horse's chest movements were counted. • Rectal temperature: recorded using a digital thermometer (range, 32–42 °C; accuracy, 0.1 °C). -Plasmapheresis: was performed by using a Hemofenix® plasmapheresis machine commercialized by Hemofenix-France utilizing Trackpore® technology (Dubna Moscow region-Russian Federation). -Blood sampling: Blood samples were obtained by jugular venipuncture and were collected in BD-Vacutainer EDTA tubes for haematological parameters and in lithium heparin’BD-Vacutainer tubes for biochemical parameters. -Haematological parameters analysis was performed by using a veterinary cell-counter automate, the Celltac VET MEK-6550® haematology (Nihon Kohden, Tomioka-Japan) and its reagents, Hemolynac·3 MEK-660I, Isotonac·4 MEK-641I, Cleanac MEK-520I (Celltac VET MEK-6550 J/K haematology, Nihon Kohden, Tomioka-Japan). -Biochemical parameters were assayed using the veterinary Skyla-VB1® automate analyzer (Lite-On Technology Corporation, Hsinchu-Taiwan) and reagent disc (Equine Panel- Product code 900-150, skylaTM VB1® reagent disc, Hsinchu-Taiwan). -Lactate concentrations were determined using Lactate-Pro™ 2 analyzer (Busimedic®, S.L, San Sebastián-Spain).
Data format	Raw Data
Parameters for data collection	The data were obtained from 12 show jumping horses that were divided into plasmapheresis (n = 6) and control (n = 6) groups. Each group contained 3 females and 3 males of Anglo-Hispano-Arab breed, aged from 4 to 12 years and weighing 400 kg. Throughout the study, horses were healthy and their clinical state was rigorously controlled.
Description of data collection	All horses underwent the same daily physical exercise for three consecutive days. Following the 3rd day exercise, a plasmapheresis session was performed only on horses of plasmapheresis group. All physiological and haemato-biochemical parameters data presented in this article were obtained from blood samples taken simultaneously on horses of the plasmapheresis and control groups before and after each exercise and after plasmapheresis session for the following 4 days.
Data source location	Royal Guard of Tétouan Tétouan Morocco Latitude: 35.5730287 Longitude: 5.3515084 GPS Coordinates: 35° 34′ 22.904″ N 5° 21′ 5.429″ W
Data accessibility	Raw Data is available with this article
Related research article	Réda Daden^a,b^, Fatima Zahraa Zarhouni^a^, Jamal Chakir^c^, Mohammed Piro^d^, Mohamed Rachid Achaaban^a^, Mohammed Ouassat^a^ & Khalid El Allali^a^. “Plasmapheresis Effect on Haematological and Biochemical Parameters in Athletic Horses Subjected To Exercise” Journal of Equine Veterinary Science https://doi.org/10.1016/j.jevs.2019.07.006

**Table:** 

**Value of the Data** • This data represents for the first time the changes in physiological, haematological and biochemical parameters in horses subjected to a plasmapheresis session after three days of exercise. It can be thus taken into consideration by other researchers interested in both sport physiology and blood filtration techniques field. • Such data can be used as a reference series for comparative approaches in the field. By providing relevant information, it allows a reuse and a better interpretation of the results. This is also useful for future studies. • In addition, the present data show informations that are complementing the related article and can be interesting for readers of the Data in Brief journal.

## 1 Data

Data shown in this article provide information about the effect of plasmapheresis on physiological, haematological and biochemical parameters in athletic horses subjected to exercise. Horses of both plasmapheresis and control groups underwent each morning an exercise of 30 min/day, during 3 successive days (Day −2, −1 and 0). Following the exercise session of the 3rd day (Day 0) a plasmapheresis session was performed only on the horses of the plasmapheresis group. The control group were maintained in the same environment but without being subjected to plasmapheresis. Data of haemato-biochemical parameters were obtained by analyzing blood samples collected before and after exercise and plasmapheresis. Table 1, Table 2, Table 3 describe clinical parameters variations subsequent to an exercise and a plasmapheresis session. Table 4, Table 5, Table 6, Table 7, Table 8, Table 9, Table 10 represent each one parameters of red hemogram before and after exercise also before and after a plasmapheresis session. Table 11, Table 12, Table 13, Table 14, Table 15 describe parameters of white hemogram and their changes subsequent to exercise and plasmapheresis session. Table 16, Table 17, Table 18, Table 19, Table 20, Table 21, Table 22, Table 23, Table 24, Table 25, Table 26, Table 27, Table 28, Table 29, Table 30, represent variations of biochemical parameters before and after graded exercise and plasmapheresis session.

**Table 1:** Rectal temperature (°C)^a^ in jumping horses subsequent to an exercise and a plasmapheresis session.

Group	Horse	Time (Days/Samples)										
		D-2		D-1		D0			D1	D2	D3	D4
		S1	S2	S3	S4	S5	S6	S7	S8	S9	S10	S11
Control Group	Acolito	37.5	40.1	37.5	40.2	37.5	40.3	38.1	37.5	37.8	37.6	37.5
	Dahbi	37.2	40	37.8	40.8	37.5	40.9	37.5	38.3	38.6	38.5	38.6
	Chamaa	37.8	40.6	37.6	40.6	37.5	40.5	38.1	37.9	37.6	37.5	37.6
	Dahman	37.6	39.9	37.6	39.9	37.6	40.1	38.2	37.5	37.5	37.5	37.5
	Damaa	37.5	39.9	37.6	40	37.6	40.1	38.3	37.6	37.5	37.5	37.6
	Dalilano	37.8	41	37.6	39.9	37.6	40.1	38.2	37.5	37.5	37.5	37.5
Plasmapheresis Group	Beluc	37.6	39.9	37.6	39.9	37.6	40.1	38.2	37.5	37.5	37.5	37.5
	Chaddy	37.5	40.8	37.5	40.9	37.5	40.9	37.5	38.3	38.6	38.5	38.6
	Printanier	37.7	40.9	37.8	40.8	37.7	40.9	37.9	37.8	37.8	37.9	37.8
	Daoudiya	37.7	40.6	37.8	41	37.8	40.9	40.1	37.9	37.8	37.8	37.8
	Camelia	37.6	40.6	37.6	40.6	37.5	40.5	38.1	37.9	37.6	37.5	37.6
	Doualiya	37.8	39.9	37.9	40.2	37.5	40.1	39.1	38.2	37.5	38.5	39.5

a Normal rectal temperature of adult horse: 37.0–38.0 °C [3].

**Table 2:** Heart rate (beats per minute)^a^ changes in jumping horses subsequent to an exercise and a plasmapheresis session.

Group	Horse	Time (Days/Samples)										
		D-2		D-1		D0			D1	D2	D3	D4
		S1	S2	S3	S4	S5	S6	S7	S8	S9	S10	S11
Control Group	Acolito	36	112	36	116	36	120	38	36	36	38	36
	Dahbi	36	120	36	126	36	123	36	36	36	36	36
	Chamaa	32	120	36	132	36	123	38	36	36	38	36
	Dahman	36	110	36	115	36	110	36	36	38	36	36
	Damaa	36	120	36	132	36	152	36	36	38	36	38
	Dalilano	36	115	36	110	36	110	36	36	38	36	36
Plasmapheresis Group	Beluc	36	110	36	113	36	100	36	36	38	36	36
	Chaddy	36	104	36	100	36	120	36	36	36	36	36
	Printanier	45	167	44	162	44	152	46	44	45	46	44
	Daoudiya	41	110	44	120	44	118	45	44	42	44	40
	Camelia	32	110	36	120	36	114	38	36	36	38	36
	Doualiya	38	112	40	132	42	110	40	36	38	38	36

a Normal heart rate of adult horse: 30–40 beats per minute [3].

**Table 3:** Respiratory rate (breaths per minute)^a^ changes in jumping horses subsequent to an exercise and a plasmapheresis session.

Group	Horse	Time (Days/Samples)										
		D-2		D-1		D0			D1	D2	D3	D4
		S1	S2	S3	S4	S5	S6	S7	S8	S9	S10	S11
Control Group	Acolito	24	68	26	68	26	68	28	24	24	24	24
	Dahbi	20	68	24	64	20	72	24	28	24	28	24
	Chamaa	20	68	24	62	20	66	24	24	20	24	24
	Dahman	20	64	28	68	24	66	28	24	22	24	24
	Damaa	20	64	28	68	24	66	28	24	22	24	24
	Dalilano	20	66	28	68	24	68	28	24	22	24	24
Plasmapheresis Group	Beluc	20	64	28	68	24	66	28	24	22	24	24
	Chaddy	24	64	20	72	24	68	24	28	24	28	24
	Printanier	28	98	32	68	28	72	24	28	28	28	28
	Daoudiya	28	64	28	63	26	68	28	26	24	24	24
	Camelia	24	68	24	63	20	68	24	24	20	24	24
	Doualiya	24	68	28	68	24	66	28	24	28	26	24

a Normal respiratory rate of adult horse: 8–20 breaths per minute [4].

**Table 4:** Red blood cell count (10^6^/μl)^a^ changes in jumping horses subsequent to exercise and a plasmapheresis session.

Group	Horse	Time (Days/Samples)										
		D-2		D-1		D0			D1	D2	D3	D4
		S1	S2	S3	S4	S5	S6	S7	S8	S9	S10	S11
Control Group	Acolito	8.88	10.6	8.58	10.4	10.2	13.2	8.01	7.47	7.92	8.62	8.71
	Dahbi	8.63	13.3	8.58	10.4	8.61	10.7	8.67	10.3	9.6	8.17	8.63
	Chamaa	8.71	12.2	9.42	11.9	11.2	14.3	10	8.49	9.11	8.56	9.01
	Dahman	7.59	12.1	9.74	11.6	9.34	11.2	10.2	7.58	7.75	7.53	7.56
	Damaa	7.59	12.1	9.74	11.6	8.02	11.9	7.99	7.58	7.75	7.53	7.56
	Dalilano	7.53	12.1	9.74	11.6	8.1	12.4	9.52	7.75	7.53	7.74	13.4
Plasmapheresis Group	Beluc	11.7	11.3	9.74	12.9	10.2	13.1	13.2	12.3	10.6	10.4	9.31
	Chaddy	11.2	15.3	9.94	14.5	11.2	14.7	13.5	12.1	10.3	9.27	10.3
	Printanier	8.26	11.4	8.3	11.5	8.35	11.3	11.9	10.3	10.2	7.96	7.43
	Daoudiya	7.56	12.1	9.42	11.9	9.79	12.6	12.8	10.2	7.96	7.82	10.2
	Camelia	8.71	12.2	9.42	12.3	10.4	12.4	12.7	8.03	6.95	7.22	10.5
	Doualiya	7.92	11.4	7.39	11.5	8.35	11.3	13	7.25	8	8.19	8.39

a Reference range: 6.70–12.90.10^6^/μL [5].

**Table 5:** Changes in blood hemoglobin concentrations (g/dl)^a^ in jumping horses subsequent to exercise and a plasmapheresis session.

Group	Horse	Time (Days/Samples)										
		D-2		D-1		D0			D1	D2	D3	D4
		S1	S2	S3	S4	S5	S6	S7	S8	S9	S10	S11
Control Group	Acolito	14.2	16.9	13.7	17.5	11.7	17.4	13	12.3	13.2	14.1	14.3
	Dahbi	13.3	19.3	13.2	16.7	13.2	16.8	13.5	15.8	14.6	12.7	13.4
	Chamaa	13	17.3	14.1	17.4	14.5	17.8	12.9	12.9	13.4	13.2	13
	Dahman	11	18.2	15.5	17.6	15.8	16.2	12.1	11.3	10.9	11	10.8
	Damaa	11	15.6	11.5	14.3	11.6	14.1	11.3	12.7	11.3	11.9	12.7
	Dalilano	11.1	18.2	15.5	17.6	15.8	18.1	11.8	10.9	11	11	18.9
Plasmapheresis Group	Beluc	17	17.7	15.5	19.4	15.3	19.8	19.6	14.6	14.8	15.6	13.8
	Chaddy	15.2	24.4	13.4	19.2	14.3	19.7	19.2	13.9	13.5	12.5	14.5
	Printanier	12.2	17.3	12.1	17	12	16.8	17	12	12.2	11.8	11.1
	Daoudiya	10.5	18	14.1	17.4	14.5	17.1	19.2	12.8	12	10.7	13.6
	Camelia	13	17.3	14.1	17.4	16.8	17.5	17.5	12.2	12.3	10.8	15.8
	Doualiya	10.8	17.3	10.3	17	12	16.8	17	12.3	11.9	12.6	12.2

a Reference range: 11.0–19.0 g/dl [6].

**Table 6:** Blood hematocrit (g/dL)^a^ changes in jumping horses subsequent to exercise and a plasmapheresis session.

Group	Horse	Time (Days/Samples)										
		D-2		D-1		D0			D1	D2	D3	D4
		S1	S2	S3	S4	S5	S6	S7	S8	S9	S10	S11
Control Group	Acolito	43.9	52.5	42.5	51.7	36.5	52.9	39.5	39.9	38	35.2	34
	Dahbi	40.4	59.8	40.4	54	40.3	50.8	41.1	44.9	39	37.7	39.5
	Chamaa	39.5	52.4	42.5	52.4	44.4	53.3	38.9	38.5	36	34.2	38.8
	Dahman	34	54.9	43.8	50	43	49	36.2	38.5	37.4	34.9	35.6
	Damaa	36.6	48.5	36.8	53	35.9	56.1	48.9	43.1	49	51.3	34.4
	Dalilano	33	54.9	42.9	52	43	54.8	36	37.7	36	37	34.2
Plasmapheresis Group	Beluc	37.5	53.3	43.8	57.9	45.7	59.2	59	31.9	35.4	33.7	35.6
	Chaddy	37.9	53	41.3	53	43	61.4	60.9	32.8	33.3	32.6	32.8
	Printanier	36.1	51.3	36.8	53	37	51.2	53	32.7	31.1	28.2	30.6
	Daoudiya	38.5	55.2	42.5	52.4	44.4	53.9	54	31.2	40.1	37.3	31.3
	Camelia	42.1	52.4	42.5	52.4	45	53	50.6	37.7	36.8	38.1	35.4
	Doualiya	37	51.3	32.8	51.4	33.8	51.2	53	34.7	36.7	33	33.8

a Reference range: 32.0–53.0 g/dl [6].

**Table 7:** Changes in blood mean corpuscular volume (fL)^a^ in jumping horses subsequent to exercise and a plasmapheresis session.

Group	Horse	Time (Days/Samples)										
		D-2		D-1		D0			D1	D2	D3	D4
		S1	S2	S3	S4	S5	S6	S7	S8	S9	S10	S11
Control Group	Acolito	44.8	46	45	46	44.6	45.2	44.8	44.6	44.4	44.5	44.6
	Dahbi	41.4	44	41.5	44.1	41.3	43	41.7	41.3	41.6	41.2	40.9
	Chamaa	44.3	45.2	44.2	45	44.3	45.2	44.9	44.4	44.1	44.3	44.5
	Dahman	42.1	45.6	45.4	45.5	45.4	43	42.7	42.3	42.2	42.2	42.2
	Damaa	45	46.8	44.6	45.5	44.7	45.2	45.6	45.3	45	45.3	45.5
	Dalilano	43.4	45.2	43	45.3	44.3	45.2	43.9	43	43.6	45.5	43.9
Plasmapheresis Group	Beluc	45.4	46	45.5	46.1	44	45.5	45.5	44	44.9	45	45
	Chaddy	46.8	47	46.3	47.4	45	46	47.1	45	44	47.2	47
	Printanier	41.4	43	41.3	45.5	45.4	45.9	45.2	45.1	44.9	45.2	44.9
	Daoudiya	44.8	45.4	45	45.6	45.2	45.5	45.3	45.1	45	45	44.7
	Camelia	41.2	42.8	40.9	42.3	41.2	42.1	42.4	41.6	41.3	41.8	41.7
	Doualiya	44.8	45.4	45	45.6	43	43.1	43.2	44.9	45	43	42.9

a Reference range: 37.0–59.0 fL [6].

**Table 8:** Changes in blood mean corpuscular hemoglobin (pg)^a^ in jumping horses subsequent to exercise and a plasmapheresis session.

Group	Horse	Time (Days/Samples)										
		D-2		D-1		D0			D1	D2	D3	D4
		S1	S2	S3	S4	S5	S6	S7	S8	S9	S10	S11
Control Group	Acolito	14.9	15.7	14.9	15.1	14.9	15.1	14.8	14.5	14.8	14.8	14.8
	Dahbi	13.5	15.4	13.8	15.5	13.1	15.2	14.5	13.8	13.6	13.5	13.2
	Chamaa	14.8	15.2	14.7	15	14.4	14.8	14.4	14.9	15	15.1	14.9
	Dahman	14.5	14.9	14.7	15.1	14.8	15.3	14.2	14	13.9	13.7	13.4
	Damaa	14.9	15	14.7	15.1	14.7	15.4	15.3	15.2	15.1	15	15.1
	Dalilano	14	15.2	13.8	15	14.4	15.2	14.7	14.6	14.1	14.6	15.1
Plasmapheresis Group	Beluc	15	16	15.2	16.2	15	16.3	16.2	15	14.9	15.2	15
	Chaddy	15.6	16.2	15.4	16.1	15.5	15.7	15.8	15.4	15.6	15.5	15.5
	Printanier	14	14.1	14	14.6	14	15.3	15.4	14.3	14.3	14.9	14.5
	Daoudiya	14.5	15	14.6	14.9	14.4	14.7	14.9	14.6	14.5	14.6	14.8
	Camelia	13.9	14.1	13.5	14.2	13.9	13.8	13.5	13.9	13.6	13.6	13.8
	Doualiya	14.5	15	14.3	15	14.5	14.6	14.8	14.5	14.6	14.2	14.1

a Reference range: 12.3–19.9 pg [7].

**Table 9:** Changes in blood mean corpuscular hemoglobin concentration (g/dL)^a^ in jumping horses subsequent to exercise and a plasmapheresis session.

Group	Horse	Time (Days/Samples)										
		D-2		D-1		D0			D1	D2	D3	D4
		S1	S2	S3	S4	S5	S6	S7	S8	S9	S10	S11
Control Group	Acolito	32.3	33	32.2	33.8	32.1	32.9	32.9	33.4	33.1	32.8	32.5
	Dahbi	32.9	33.3	32.7	33.9	32.8	33.1	32.8	32.8	33.2	33.1	33
	Chamaa	32.9	33	32.8	33.1	32.7	33.4	33.1	32.5	32.8	32.8	32.9
	Dahman	32.4	33.2	32.3	33.1	32.4	33.1	33.4	32.7	32.5	32.6	32.6
	Damaa	32.9	33	32.8	33.1	32.7	33.4	33.2	32.5	32.1	32	33
	Dalilano	32.4	33.2	32.3	33.1	32.4	33	33	32.9	33	33	32.6
Plasmapheresis Group	Beluc	32.4	33.2	32.5	33.5	32.5	33.4	33.1	32.4	32.3	32	32.5
	Chaddy	32.7	33.2	33.2	33.2	32.6	33.1	33.6	33.6	33	32.7	32.1
	Printanier	33.3	33.7	32.8	33.5	32.4	33.2	32.2	32.1	32	32.5	33.1
	Daoudiya	33	32.6	32.8	33.1	32.7	33.1	31.6	32.1	32	32.4	32.6
	Camelia	32.9	33	32.8	33.1	32.2	33.2	32.4	32.6	32.5	32.6	32.5
	Doualiya	32.3	33.7	32.1	33.5	32.4	33.6	33.2	32	32.9	33	33.2

a Reference range: 31–38.6 g/dl [7].

**Table 10:** Blood platelet count (10^3^/μl)^a^ variations in jumping horses subsequent to exercise and a plasmapheresis session.

Group	Horse	Time (Days/Samples)										
		D-2		D-1		D0			D1	D2	D3	D4
		S1	S2	S3	S4	S5	S6	S7	S8	S9	S10	S11
Control Group	Acolito	172	179	171	193	155	173	164	143	158	152	148
	Dahbi	152	146	146	165	148	145	149	135	139	144	135
	Chamaa	125	170	136	198	134	164	133	147	158	149	153
	Dahman	143	127	143	119	141	119	131	150	152	148	151
	Damaa	152	170	149	198	134	180	128	139	147	156	163
	Dalilano	143	127	143	119	141	114	95	152	148	133	77
Plasmapheresis Group	Beluc	144	162	143	134	144	122	138	141	159	170	136
	Chaddy	154	154	153	157	156	140	156	150	150	104	150
	Printanier	159	173	161	173	145	171	150	144	148	155	157
	Daoudiya	145	151	136	198	134	157	151	151	142	143	110
	Camelia	125	170	136	198	127	186	11	109	111	114	73
	Doualiya	157	173	179	173	145	171	174	157	168	194	135

a Reference range: 100–270 10^3^/μL [5].

**Table 11:** White blood cell count (10^3^/μl)^a^ changes in jumping horses subsequent to exercise and a plasmapheresis session.

Group	Horse	Time (Days/Samples)										
		D-2		D-1		D0			D1	D2	D3	D4
		S1	S2	S3	S4	S5	S6	S7	S8	S9	S10	S11
Control Group	Acolito	8.7	10.4	8.4	9.9	9.1	9.5	11.8	8.5	7.9	8.1	8
	Dahbi	5.6	8.1	6.3	7.7	8.2	10	7.6	5.8	5.7	5.6	5.1
	Chamaa	10.8	14.2	10.6	13.9	10.2	13.2	14.5	11.1	10.8	10.7	11
	Dahman	8	10.1	8.4	12.1	8.5	11	12.4	9.3	8.9	8.6	7.2
	Damaa	7.9	10.6	8.6	12.6	10.2	10.5	7.6	8.4	7.6	7.8	7.9
	Dalilano	8.1	10.1	8.4	12.1	10	8.9	9.9	8.9	8.6	10.2	5.9
Plasmapheresis Group	Beluc	7.8	7.5	8.4	7.7	9	8.1	12.6	8.7	7.8	7.6	7.6
	Chaddy	11.1	15.3	10.6	13.4	11	16.4	16.8	14.5	11.3	10.1	11.3
	Printanier	9.3	11.7	9.4	11.6	9.9	11.8	12	9.4	8.8	8.2	7.8
	Daoudiya	8.8	13.8	10.6	13.9	10.7	12.3	12.5	10.3	9.8	8.1	5.8
	Camelia	10.8	14.2	10.6	13.9	10.4	14.3	14.6	9	8.6	7.8	6.9
	Doualiya	9.4	11.7	9.7	11.6	9.9	11.8	12.4	9.3	9.7	9.3	9

a Reference range: 5.4–14.3 10^3^/μL [5].

**Table 12:** Changes in blood lymphocyte count (10^3^/μl)^a^ in jumping horses subsequent to exercise and a plasmapheresis session.

Group	Horse	Time (Days/Samples)										
		D-2		D-1		D0			D1	D2	D3	D4
		S1	S2	S3	S4	S5	S6	S7	S8	S9	S10	S11
Control Group	Acolito	6.9	8.2	6.3	8.9	7.9	9.6	7.3	7.2	7	6.5	6.4
	Dahbi	5.2	10.6	5.6	10.3	6.4	9.1	8.3	6.2	5.5	5.6	5.7
	Chamaa	6.9	10.9	6.3	10.3	6.8	7.2	10.1	6.2	7.5	6.4	6.2
	Dahman	5.9	10.6	5.8	11.5	5.9	9.6	9.8	7.5	7.2	5.8	5.9
	Damaa	5.3	9.3	5.4	10.3	7.4	9.2	8.6	6.5	6.3	5.9	5.5
	Dalilano	5.8	9.5	6.3	6.3	6.2	9.4	9.3	7.5	6.2	5.5	6.2
Plasmapheresis Group	Beluc	4	7.1	6.6	7.9	8.3	8.6	8.8	7.5	6.2	5.7	5.6
	Chaddy	12.5	9.9	5.9	9.7	6.3	6	7.2	7.9	7.8	6	6.5
	Printanier	5.9	8.8	5.8	9	7.3	10.6	10.8	8.5	8.1	7.8	6
	Daoudiya	6.9	9.7	6.4	9.6	7.3	9.6	10.2	8.1	7.5	7.2	6.8
	Camelia	6.4	13.1	6.4	9.1	7.2	10.6	10.9	7.2	6.9	6.8	6.3
	Doualiya	5.9	9.2	5.8	10	7.9	12	12.6	7	6.9	6.8	5.8

a Reference range: 1.5–7.7 10^3^/μl [6].

**Table 13:** Variations of blood Monocyte count (10^3^/μl)^a^ in jumping horses subsequent to exercise and a plasmapheresis session.

Group	Horse	Time (Days/Samples)										
		D-2		D-1		D0			D1	D2	D3	D4
		S1	S2	S3	S4	S5	S6	S7	S8	S9	S10	S11
Control Group	Acolito	0.2	0.3	0.2	0.2	0.2	0.3	0.4	0.2	0.2	0.3	0.4
	Dahbi	0.3	0.3	0.2	0.3	0.2	0.3	0.2	0.2	0.1	0.3	0.1
	Chamaa	0.3	0.3	0.3	0.4	0.3	0.5	0.3	0.2	0.2	0.1	0.2
	Dahman	0.2	0.4	0.3	0.5	0.2	0.4	0.2	0.3	0.3	0.1	0.2
	Damaa	0.3	0.3	0.3	0.4	0.3	0.4	0.3	0.2	0.2	0.2	0.3
	Dalilano	0.2	0.4	0.3	0.5	0.2	0.5	0.2	0.3	0.3	0.3	0.1
Plasmapheresis Group	Beluc	0.2	0.3	0.1	0.3	0.2	0.4	0.4	0.2	0.3	0.3	0.4
	Chaddy	0.1	0.3	0.1	0.3	0.2	0.6	0.5	0.4	0.3	0.1	0.4
	Printanier	0.3	0.2	0.2	0.2	0.3	0.3	0.2	0.6	0.2	0.3	0.1
	Daoudiya	0.2	0.4	0.2	0.3	0.3	0.3	0.4	0.2	0.3	0.3	0.1
	Camelia	0.3	0.3	0.4	0.3	0.2	0.4	0.6	0.1	0.2	0.2	0.2
	Doualiya	0.2	0.2	0.2	0.4	0.2	0.3	0.2	0.2	0.2	0.2	0.2

a Reference range: 0–0.8 10^3^/μl [8].

**Table 14:** Blood Eosinophil count (10^3^/μl)^a^ changes in jumping horses subsequent to exercise and a plasmapheresis session.

Group	Horse	Time (Days/Samples)										
		D-2		D-1		D0			D1	D2	D3	D4
		S1	S2	S3	S4	S5	S6	S7	S8	S9	S10	S11
Control Group	Acolito	0.2	0.2	0.1	0.2	0.1	0.6	0.4	0.2	0.2	0.1	0.1
	Dahbi	0.3	0.2	0.4	0.4	0.3	0.9	0.5	0.1	0.2	0.3	0.3
	Chamaa	0.3	1.3	0.3	0.9	0.3	0.7	0.3	0.3	0.3	0.2	0.1
	Dahman	0.3	0.6	0.1	0.4	0.3	0.9	0.5	0.3	0.3	0.2	0.3
	Damaa	0.3	1.3	0.3	0.9	0.3	0.8	0.5	0.4	0.4	0.3	0.2
	Dalilano	0.2	0.6	0.2	0.4	0.3	0.9	0.6	0.2	0.2	0.2	0.1
Plasmapheresis Group	Beluc	0.3	0.4	0.3	0.1	0.3	0.6	0.1	0.3	0.1	0	0
	Chaddy	0.1	0.9	0.2	0.7	0.2	1.6	1.5	0.5	0.4	0.3	0.2
	Printanier	0.4	0.8	0.3	0.9	0.3	0.8	0.9	0.4	0.1	0.3	0.5
	Daoudiya	0.4	1.4	0.3	0.9	0.3	0.8	0.9	0.4	0.6	0.5	0.3
	Camelia	0.3	1.3	0.3	0.9	0.4	1	0.8	0.3	0.7	0.2	0.3
	Doualiya	0.2	0.9	0.4	0.9	0.5	0.8	0.9	0.4	0.4	0.9	0.2

a Reference range: 0–1.0 10^3^/μl [6].

**Table 15:** Blood Granulocyte count (10^3^/μl)^a^ variations in jumping horses subsequent to exercise and a plasmapheresis session.

Group	Horse	Time (Days/Samples)										
		D-2		D-1		D0			D1	D2	D3	D4
		S1	S2	S3	S4	S5	S6	S7	S8	S9	S10	S11
Control Group	Acolito	5.9	6.1	5.1	9.6	5.4	5.7	6.1	5.4	5.5	5	5.3
	Dahbi	3.2	5.3	3	5.8	1.9	2.6	3	2.6	2.9	0.5	2.6
	Chamaa	6.1	6.2	3.5	8.6	6.3	6.4	6.4	6.4	6.2	5.7	6.3
	Dahman	3.7	3.6	3.5	8.5	5.3	6.8	5.2	4.8	4.9	3.6	3.4
	Damaa	7.9	10.6	8.6	7.6	7.6	12.6	8.4	7.8	6.5	10.5	7.9
	Dalilano	3.7	3.6	6.1	3.3	5.3	6.8	4.9	3.8	4.5	2.1	2
Plasmapheresis Group	Beluc	2.8	5.2	5.2	6.3	6.3	6.1	6.5	6.3	4.6	4.3	5.1
	Chaddy	2.8	7.4	2.5	6.5	3.4	7	7.3	3.5	3.4	3.5	3.2
	Printanier	4.6	5.2	5.7	6.3	5.1	6.5	6.3	5.1	4.8	4.2	4.1
	Daoudiya	5.5	6.3	5.1	6.4	6.3	6.5	6.5	6.6	5.4	4.3	2.8
	Camelia	6.2	7.5	6.5	7.2	3.4	7.3	7.5	4.7	4.3	4	3.2
	Doualiya	4.6	6.5	5.7	6.8	5.1	6.2	6.4	5	5.1	5	4.6

a Reference range: 2.3–8.6 10^3^/μl [7].

**Table 16:** Blood glucose concentration (mmol/L)^a^ in jumping horses submitted to plasmapheresis after graded exercise.

Group	Horse	Time (Days/Samples)										
		D-2		D-1		D0			D1	D2	D3	D4
		S1	S2	S3	S4	S5	S6	S7	S8	S9	S10	S11
Control Group	Acolito	5.2	6	4.5	6	4.5	5.6	5.7	5.7	5.6	5.5	5.6
	Dahbi	7.2	6	4.9	5.8	4.8	6.8	5.3	5.7	5.4	5.3	5.2
	Chamaa	5.5	7.1	6.2	5.8	5.3	6.9	6.2	5.8	5.3	5.6	5.9
	Dahman	4.6	5.6	4.5	5.7	5.3	6.3	5.7	5.4	5.3	4.9	4.8
	Damaa	5.5	7.1	6.2	8.2	6.6	6.2	5.8	5.6	5.3	5.6	6.2
	Dalilano	4.6	5.6	4.5	5.7	5.3	6.3	5.1	4.8	5.3	4.9	5.4
Plasmapheresis Group	Beluc	4.8	5.6	5.3	6.3	5.4	5.2	5.4	5.2	5.1	4.9	4.6
	Chaddy	5.6	5.8	4.9	6.6	4.9	9.3	5.5	5.5	5.4	4.9	4.6
	Printanier	3.8	6.5	3.6	4.5	4.5	6.4	5.7	4.8	4.7	4.6	4.6
	Daoudiya	5.5	7.1	6	6.3	6.1	6.3	5.5	6.8	6.7	5.9	5.6
	Camelia	7.8	5.3	5.5	5.6	5.4	5.4	3.6	4.8	4.7	4.8	4.7
	Doualiya	5.8	7.1	5.9	8.2	5.6	5.8	5.9	5	4.7	4.6	5.5

a Reference range: 3.5–6.0 mmol/L [9].

**Table 17:** Modifications of blood urea concentrations (mmol/L)^a^ in jumping horses subsequent to exercise and a plasmapheresis session.

Group	Horse	Time (Days/Samples)										
		D-2		D-1		D0			D1	D2	D3	D4
		S1	S2	S3	S4	S5	S6	S7	S8	S9	S10	S11
Control Group	Acolito	4.1	4.2	4.1	4.1	3.8	3.9	3.9	3.8	3.9	3.8	3.6
	Dahbi	4.8	5.3	4.9	5.4	5.1	5.5	5.5	5.6	5.1	4.9	4.7
	Chamaa	5.3	5.9	5.6	6.3	6	6.2	5.8	5.6	5.3	4.6	4.7
	Dahman	5	5.4	5.2	5.8	5.3	5.6	5.3	5.4	5.6	4.9	4.6
	Damaa	3.9	4.1	4	4.2	4.5	5	4	3.9	3.8	3.7	3.5
	Dalilano	5	5.4	5.1	5.8	5	5.7	4.9	4.9	4.8	4.7	4.6
Plasmapheresis Group	Beluc	4.3	4.9	4.6	4.7	4	4.2	3.6	3.2	3.8	3.7	3.2
	Chaddy	3.9	3.9	4	4.3	3.8	3.9	3.2	3.5	3.2	3	3.1
	Printanier	4.5	4.6	4.8	5	5.1	5.8	4.9	4	4.1	4.1	4
	Daoudiya	3.8	4.1	3.9	4.2	4.6	5.3	5	5.1	5.3	4.2	3.1
	Camelia	6	6.5	6	6.5	6.3	7.8	6.3	5.6	5.4	5.6	5.6
	Doualiya	5.1	5.2	5.1	5.2	4.9	4.6	4.3	4.2	4	4.1	4

a Reference range: 1.2 à 3.6 mmol/L [9].

**Table 18:** Changes in blood creatinine concentrations (mg/dL)^a^ in jumping horses subsequent to exercise and a plasmapheresis session.

Group	Horse	Time (Days/Samples)										
		D-2		D-1		D0			D1	D2	D3	D4
		S1	S2	S3	S4	S5	S6	S7	S8	S9	S10	S11
Control Group	Acolito	1.9	2.2	1.9	2	2	2.4	2	1.8	1.9	2	2.1
	Dahbi	1.7	1.9	1.8	2.1	2	2.3	2.1	2	2.3	2.1	2
	Chamaa	1.2	1.7	1.2	1.9	1.3	1.8	1.3	1.8	1.7	1.5	1.4
	Dahman	1.8	2.5	1.9	2.4	1.8	1.9	2	1.6	1.7	1.8	1.6
	Damaa	1.4	1.7	1.4	1.9	1.5	2	2.2	1.8	1.7	1.5	1.6
	Dalilano	1.8	2.5	1.9	2.4	2.1	2.2	2.1	1.8	1.7	1.8	1.5
Plasmapheresis Group	Beluc	1.6	2.3	1.7	2	1.6	1.9	1.8	1.8	1.7	1.8	1.8
	Chaddy	1.5	1.5	1.5	1.8	1.6	2	1.7	1.6	1.6	1.5	1.4
	Printanier	1.7	2.3	1.5	2.3	1.8	2.2	1.8	1.7	1.8	1.7	1.8
	Daoudiya	1.3	1.7	1.3	1.9	1.4	2.3	1.9	1.6	1.7	1.6	1.6
	Camelia	1.4	1.6	1.7	1.9	1.5	1.5	1.4	1.5	1.3	1.4	1.3
	Doualiya	1.6	1.7	1.5	1.9	1.5	1.5	1.4	1.4	1.8	1.7	1.5

a Reference range: 0.8–1.5 mg/dl [10].

**Table 19:** Blood Alkaline phosphatase concentrations (U/L)^a^ in jumping horses subsequent to exercise and a plasmapheresis session.

Group	Horse	Time (Days/Samples)										
		D-2		D-1		D0			D1	D2	D3	D4
		S1	S2	S3	S4	S5	S6	S7	S8	S9	S10	S11
Control Group	Acolito	158	162	141	184	145	174	174	159	158	159	160
	Dahbi	84	98	88	88	86	112	102	96	95	93	93
	Chamaa	117	141	127	144	140	154	150	132	139	140	141
	Dahman	110	124	141	142	140	142	130	129	123	110	101
	Damaa	117	141	127	144	140	136	143	141	139	140	122
	Dalilano	110	112	112	142	111	125	124	124	123	110	118
Plasmapheresis Group	Beluc	100	104	140	156	135	140	164	137	136	132	129
	Chaddy	131	139	88	156	136	177	141	141	132	129	121
	Printanier	110	113	111	117	115	119	117	112	111	112	110
	Daoudiya	117	141	127	144	140	147	123	111	111	110	110
	Camelia	161	139	160	142	159	140	148	171	165	169	146
	Doualiya	116	141	127	144	140	126	133	123	111	112	110

a Reference value: < 250U/L [5].

**Table 20:** Blood Gama Glutamyl Transferase concentrations (U/L)^a^ in jumping horses subsequent to exercise and a plasmapheresis session.

Group	Horse	Time (Days/Samples)										
		D-2		D-1		D0			D1	D2	D3	D4
		S1	S2	S3	S4	S5	S6	S7	S8	S9	S10	S11
Control Group	Acolito	10	10	10	10	10	10	10	10	10	10	10
	Dahbi	10	10	10	10	10	10	10	10	10	10	10
	Chamaa	11	14	15	18	18	22	17	16	10	12	13
	Dahman	10	10	10	10	10	10	10	10	10	10	10
	Damaa	11	14	15	18	18	11	10	10	10	12	10
	Dalilano	10	10	10	10	10	13	10	10	10	10	10
Plasmapheresis Group	Beluc	10	10	10	10	10	10	10	10	10	10	10
	Chaddy	10	10	10	10	10	15	10	13	12	12	12
	Printanier	10	10	11	12	10	13	10	10	10	10	10
	Daoudiya	11	14	15	18	18	13	14	11	11	10	10
	Camelia	10	12	13	11	12	10	10	10	11	11	10
	Doualiya	11	10	10	18	18	11	11	10	10	10	10

a Reference value: <30 U/l [5].

**Table 21:** Blood Total bilirubin concentrations (mg/dL)^a^ in jumping horses subsequent to exercise and a plasmapheresis session.

Group	Horse	Time (Days/Samples)										
		D-2		D-1		D0			D1	D2	D3	D4
		S1	S2	S3	S4	S5	S6	S7	S8	S9	S10	S11
Control Group	Acolito	2.2	2.5	1.9	2.5	2.4	2.3	2.3	2.2	2.2	2.4	2.3
	Dahbi	1.7	3.8	1.6	1.7	1.6	1.6	2.3	2.1	2	1.8	1.3
	Chamaa	1.2	1.7	1.2	1.5	1.2	1.2	1.3	1.6	1.7	1.5	1.4
	Dahman	1.6	1.8	1.9	2.6	2.2	3.2	1.8	1.9	1.5	1.6	1.4
	Damaa	1.2	1.7	1.2	1.5	1.4	1.2	2.6	2.3	1.7	1.5	1.7
	Dalilano	1.6	1.8	1.9	2.6	1.6	3.2	1.9	1.2	1.5	1.6	1.2
Plasmapheresis Group	Beluc	3.1	1.9	3.2	3.7	3.5	3.2	3.1	2.6	2.1	2.3	1.5
	Chaddy	1.7	1.9	1.6	1.8	1.6	2	2.1	1.6	1.7	1.8	1.6
	Printanier	0.7	2.1	1.9	2	2.1	2.1	2.2	1.7	1.6	1.5	0.8
	Daoudiya	1.2	2	1.2	1.5	1.2	1.7	1.8	1.7	1.6	1.3	1.1
	Camelia	1.6	2.3	1.4	1.6	1.5	1.7	1.6	1.6	1.6	1.5	1.8
	Doualiya	1.6	2.45	1.5	1.5	1.2	1.6	1	1.7	1.7	1.7	1.6

a Reference value: 0.5–2.1 mg/dl [10].

**Table 22:** Blood Sodium concentrations (mmol/L)^a^ in jumping horses subsequent to exercise and a plasmapheresis session.

Group	Horse	Time (Days/Samples)										
		D-2		D-1		D0			D1	D2	D3	D4
		S1	S2	S3	S4	S5	S6	S7	S8	S9	S10	S11
Control Group	Acolito	138	143	138	144	143	143	138	138	142	143	144
	Dahbi	146	139	139	139	138	144	140	138	143	145	144
	Chamaa	138	146	142	153	142	146	140	138	142	146	144
	Dahman	138	143	138	142	139	149	139	138	129	121	123
	Damaa	138	146	142	153	142	149	138	146	142	146	142
	Dalilano	138	151	138	142	136	140	139	139	136	138	140
Plasmapheresis Group	Beluc	136	146	146	146	142	147	148	144	141	139	133
	Chaddy	143	142	139	144	139	146	150	146	143	139	137
	Printanier	145	149	144	145	146	145	149	145	145	149	150
	Daoudiya	138	145	142	146	145	148	148	147	142	139	138
	Camelia	142	145	140	146	139	145	147	146	143	140	148
	Doualiya	148	146	141	146	146	144	147	146	145	149	141

a Reference range: 132–142 mmol/L [11,12].

**Table 23:** Blood Potassium concentrations (mmol/L)^a^ in jumping horses submitted to plasmapheresis after graded exercise.

Group	Horse	Time (Days/Samples)										
		D-2		D-1		D0			D1	D2	D3	D4
		S1	S2	S3	S4	S5	S6	S7	S8	S9	S10	S11
Control Group	Acolito	4.4	4.6	4.3	4.7	4.2	4.7	4.6	4.5	4.7	3.5	3.1
	Dahbi	4.4	4.7	4.4	4.5	4.1	4.3	4.1	4.3	4.4	4.5	4.8
	Chamaa	3.6	4.3	4.1	4.2	4.4	4.8	4.3	3.9	3	3.2	3.3
	Dahman	4.2	4.6	4.5	4.6	4.7	4.9	4.5	3.5	3.5	3.8	3.9
	Damaa	3.6	4.5	4.1	4.2	4.4	5.2	5.1	4.6	4.6	4.5	3.1
	Dalilano	4.2	4.6	4.5	4.6	4.7	5.1	5	4.8	3	3.2	3
Plasmapheresis Group	Beluc	4.4	4.7	4.7	4.6	4.2	4.9	4.1	4.9	4.3	4.2	4.2
	Chaddy	3.9	4.3	4.2	4.1	3.9	4.8	4.1	4.3	4.2	4.4	4.5
	Printanier	4.2	4.8	5.3	5.1	4.8	5.2	4.1	3.1	3.6	3.2	2.7
	Daoudiya	3.8	4.6	4.2	4.6	4.4	5.5	4.2	3.4	3.3	3.3	2.2
	Camelia	4	4.6	3.6	4.3	4.2	3.5	3.6	4.7	3.6	3.6	3.8
	Doualiya	4.2	4.5	4.2	4.2	4.4	4.4	4.1	3.1	3.3	3.2	4.1

a Reference range: 3.8–5.2 mmol/L [9].

**Table 24:** Blood Calcium concentrations (mg/dL)^a^ in jumping horses subsequent to exercise and a plasmapheresis session.

Group	Horse	Time (Days/Samples)										
		D-2		D-1		D0			D1	D2	D3	D4
		S1	S2	S3	S4	S5	S6	S7	S8	S9	S10	S11
Control Group	Acolito	11	12	11.6	12	11.3	12.2	11.9	11.3	11.8	11.9	11.9
	Dahbi	11.9	12	11	11.9	11.3	11	11.6	11.9	11.6	11.7	11.8
	Chamaa	11.3	12	11.6	12.1	11.2	11.8	10.9	11.6	11.5	11.9	12
	Dahman	11.3	12.6	11.2	11.6	11.4	12.3	11.6	11.1	11.8	11.3	11.3
	Damaa	11.9	12.3	11.6	12.1	11.3	12.2	11.9	11.8	11.5	11.9	11.2
	Dalilano	11.3	11.5	11	11.4	11.4	11.7	11.3	11.6	11.8	11.3	11.4
Plasmapheresis Group	Beluc	11.7	12	11.6	12	11.6	11.9	11.5	11.5	11.5	11.6	11.7
	Chaddy	11.3	11.6	11.3	12	11.5	12.3	10.4	10.6	10.5	11.2	11.3
	Printanier	11	12.2	11	12.3	11.4	11.9	11.3	11.8	11.8	10.6	10.8
	Daoudiya	11.5	12	11.2	12.1	11.3	12.3	11.4	11.9	11.6	11.5	11.2
	Camelia	10.5	11.3	10.8	12.3	11.1	12	10.9	10.7	10.8	10.6	11.2
	Doualiya	11.3	12	11.2	12.3	11.3	12.3	10.3	11.6	11	11.8	11.2

a Reference range: 10.8–12.9 mg/dL [10].

**Table 25:** Plasma albumin (g/L)^a^ changes in jumping horses subsequent to exercise and a plasmapheresis session.

Group	Horse	Time (Days/Samples)										
		D-2		D-1		D0			D1	D2	D3	D4
		S1	S2	S3	S4	S5	S6	S7	S8	S9	S10	S11
Control Group	Acolito	34	37	32	37	33	36	36	33	34	35	33
	Dahbi	36	41	34	37	36	38	38	35	33	33	34
	Chamaa	35	39	36	37	38	38	38	38	33	35	37
	Dahman	32	39	32	38	33	39	37	36	35	37	29
	Damaa	35	39	36	37	38	38	35	37	36	36	35
	Dalilano	33	39	32	38	33	38	36	32	35	36	30
Plasmapheresis Group	Beluc	34	38	35	38	35	36	30	34	35	36	34
	Chaddy	36	38	34	39	35	42	32	36	36	38	33
	Printanier	31	34	32	35	32	36	26	32	31	32	32
	Daoudiya	35	39	36	37	38	39	28	35	34	33	34
	Camelia	36	32	37	30	35	31	26	34	35	32	32
	Doualiya	31	39	29	37	38	33	25	31	31	32	32

a Reference range: 26–37g/L [5].

**Table 26:** Plasma globulin (g/L)^a^ changes in jumping horses subsequent to exercise and a plasmapheresis session.

Group	Horse	Time (Days/Samples)										
		D-2		D-1		D0			D1	D2	D3	D4
		S1	S2	S3	S4	S5	S6	S7	S8	S9	S10	S11
Control Group	Acolito	33	36	31	35	31	36	33	32	33	34	35
	Dahbi	31	38	29	30	31	35	31	31	32	34	32
	Chamaa	34	37	33	35	33	38	39	34	33	32	33
	Dahman	28	37	31	35	34	36	38	35	34	36	37
	Damaa	34	37	33	35	33	38	39	33	32	33	32
	Dalilano	28	37	31	35	34	38	32	33	32	32	33
Plasmapheresis Group	Beluc	34	35	34	38	33	30	28	30	33	36	31
	Chaddy	25	42	29	46	36	47	23	28	29	26	29
	Printanier	30	33	33	35	30	35	26	30	34	33	34
	Daoudiya	34	37	33	35	33	38	32	30	29	27	26
	Camelia	30	32	32	31	39	32	27	39	34	33	35
	Doualiya	34	37	36	35	33	41	32	34	29	27	28

a Reference range: 25–45 g/L [5].

**Table 27:** Changes in Plasma total protein concentrations (g/L)^a^ in jumping horses subsequent to exercise and a plasmapheresis session.

Group	Horse	Time (Days/Samples)										
		D-2		D-1		D0			D1	D2	D3	D4
		S1	S2	S3	S4	S5	S6	S7	S8	S9	S10	S11
Control Group	Acolito	68	73	63	72	64	72	70	67	63	62	63
	Dahbi	74	79	73	67	73	73	72	71	71	71	71
	Chamaa	72	76	71	76	71	76	76	73	73	69	70
	Dahman	64	76	65	78	65	75	72	66	67	68	68
	Damaa	70	76	74	76	70	76	70	73	74	73	73
	Dalilano	66	76	66	78	65	74	70	67	62	64	63
Plasmapheresis Group	Beluc	68	73	69	76	68	66	66	67	67	65	69
	Chaddy	80	80	63	85	65	89	64	66	68	69	65
	Printanier	61	67	64	68	62	71	70	72	68	70	74
	Daoudiya	69	76	74	76	71	77	68	70	72	68	69
	Camelia	75	62	76	61	74	63	64	73	68	74	73
	Doualiya	65	76	65	76	71	74	63	66	68	68	68

a Reference range: 62.5–75.0 g/L [13].

**Table 28:** Changes in blood aspartate Amino-transferase concentrations (U/L)^a^ in jumping horses subsequent to exercise and a plasmapheresis session.

Group	Horse	Time										
		D-2		D-1		D0			D1	D2	D3	D4
		S1	S2	S3	S4	S5	S6	S7	S8	S9	S10	S11
Control Group	Acolito	242	275	276	285	283	304	302	287	279	275	274
	Dahbi	311	350	293	331	350	359	354	338	336	334	332
	Chamaa	310	365	352	388	365	391	380	378	344	345	348
	Dahman	192	251	254	263	255	267	282	256	246	232	226
	Damaa	310	365	375	388	345	352	370	369	355	345	334
	Dalilano	192	251	259	263	350	362	378	380	360	346	325
Plasmapheresis Group	Beluc	165	348	212	215	219	223	230	219	230	223	240
	Chaddy	231	245	275	289	296	309	221	227	233	236	220
	Printanier	300	310	325	349	358	359	295	315	321	305	318
	Daoudiya	310	365	372	388	376	387	289	299	304	301	302
	Camelia	357	319	359	312	345	355	201	221	232	226	210
	Doualiya	335	365	375	388	389	400	230	238	256	306	235

a Reference range: 160–412 U/L [11,12].

**Table 29:** Variations of blood creatine kinase concentrations (U/L)^a^ in jumping horses subsequent to exercise and a plasmapheresis session.

Group	Horse	Time (Days/Samples)										
		D-2		D-1		D0			D1	D2	D3	D4
		S1	S2	S3	S4	S5	S6	S7	S8	S9	S10	S11
Control Group	Acolito	165	180	172	182	136	135	274	212	182	174	169
	Dahbi	150	160	151	168	152	175	165	166	165	165	153
	Chamaa	149	230	156	268	179	231	133	154	150	136	152
	Dahman	112	210	212	250	172	205	189	212	201	196	189
	Damaa	149	169	156	162	179	201	200	189	169	156	146
	Dalilano	123	215	212	262	172	250	212	236	226	212	189
Plasmapheresis Group	Beluc	120	160	125	189	153	159	105	111	110	110	112
	Chaddy	122	201	191	203	135	183	109	102	112	120	121
	Printanier	123	151	128	159	132	149	110	103	114	117	120
	Daoudiya	123	146	156	156	179	196	123	102	123	120	120
	Camelia	306	315	302	332	151	220	132	103	114	116	123
	Doualiya	149	212	146	232	179	202	132	123	112	115	123

a Reference range: 60–330 U/L [11,12].

**Table 30:** Changes in blood lactate concentrations (mmol/L)^a^ in jumping horses subsequent to exercise and a plasmapheresis session.

Group	Horse	Time (Days/Samples)										
		D-2		D-1		D0			D1	D2	D3	D4
		S1	S2	S3	S4	S5	S6	S7	S8	S9	S10	S11
Control Group	Acolito	1	13.7	1.1	13.5	1	11.4	2.4	1	1	1	1
	Dahbi	1.2	15.2	1.2	14.2	1.3	12.6	2.2	1.2	1.3	1.2	1.3
	Chamaa	1.1	15.4	1	16.9	1	17.8	3.4	1.1	1.1	1	1.1
	Dahman	1	14.8	1	14.6	1	14.7	3.1	1.1	1	1	1.1
	Damaa	1.1	18.2	1.1	19.6	1.2	20.9	4.1	1.2	1.1	1.1	1.1
	Dalilano	1	18.6	1.1	20.1	1.1	19.7	3.9	1.1	1.1	1	1.1
Plasmapheresis Group	Beluc	1	13.2	1	13.3	1	14.5	1.1	1	0.9	0.9	0.9
	Chaddy	1.1	13.5	1	16	1	14.3	1.2	1.1	1.1	1.1	1
	Printanier	1	23.4	1.3	21.9	1.2	24.5	1.3	1.1	1.1	0.9	0.9
	Daoudiya	1	14.7	1	14.9	1	15.1	1.1	1	0.8	0.8	0.9
	Camelia	1.2	19.6	1.1	20.8	1	21.1	1.1	1.1	1	1	1
	Doualiya	1.2	17.9	1.1	18.1	1.2	18.4	1.1	1	0.9	0.8	0.9

a Reference value: < 2.5 mmol/L [9].

**Fig. 1:**
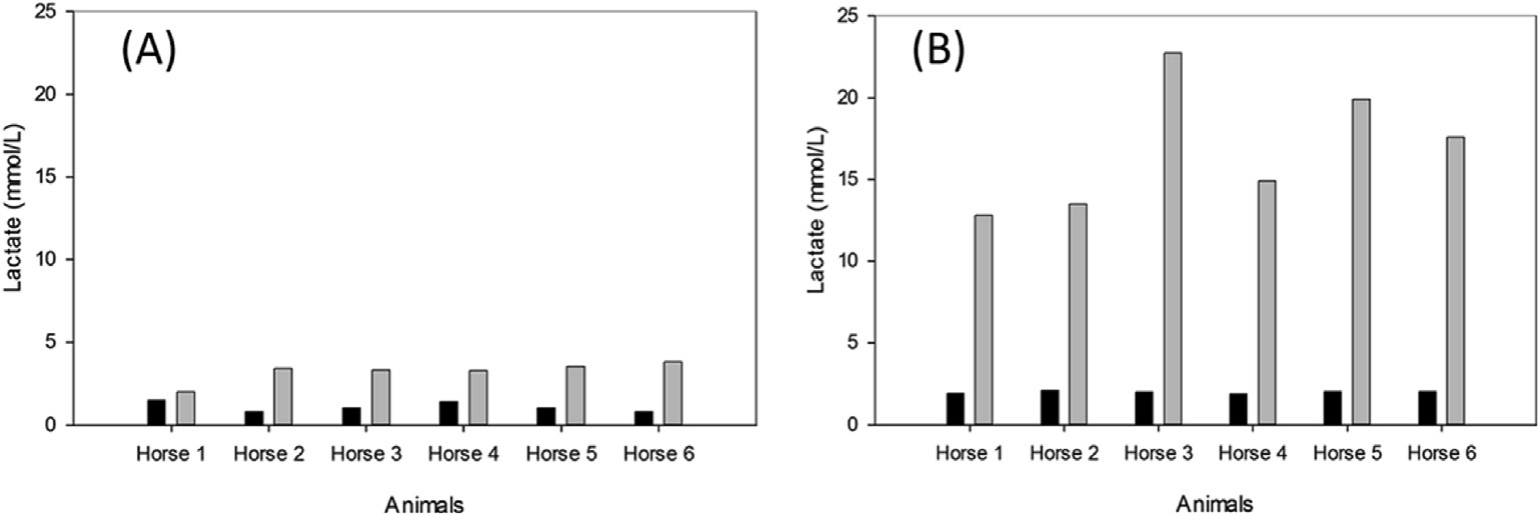
**Lactate concentrations in six jumping horses**. **(A)**: Before and following the exercise test standardized by Demonceau and Auvinet. **(B)**: Before and following the modification of the warm-up phase of the same exercise test. The duration of this phase was doubled to 30 min instead of 15 min and steps were substituted by gallop.

Fig. 1 illustrate Lactate concentrations before and following the exercise test standardized by Demonceau and Auvinet [2], also before and following the modification of the warm-up phase of the same exercise test.

### 1.1 Tables

The following tables containing raw data concerning changes in physiological, heamtological and biochemical parameters of horses subjected to graded physical exercise and plasmapheresis session. Data of each sample are presented individually for animals of the control and plasmapheresis groups. S1, S3, S5: measurements before the exercise; S2, S4, S6: measurements after the exercise; S7: immediate measurement after the plasmapheresis session; S8, S9, S10, S11: post-plasmapheresis measurements at rest. D-2: 48h before plasmapheresis D-1: 24h before plasmapheresis; Day 0: day of plasmapheresis subsequent to the last exercise; D1, D2, D3 and D4 correspond to 24h, 48h, 72h and 96h respectively after plasmapheresis.

## 2 Experimental design, materials, and methods

Investigations were carried out on twelve show jumping horses randomly allocated to two groups: a plasmapheresis group (n = 6) and a control group (n = 6). All horses were healthy and received the same food diet three times a day. First in the morning, they were offered each 1 kg of commercial feed (Destrier, France). Then, in midday, they received each 2.5 kg of barley and its bran (0.5 kg) in addition to 30 g of a vitamin and mineral supplement. Finally, in the evening, horses were fed each 0.5 kg of oats and 2 kg of barley. A supplement of 2.5 kg of alfalfa hay was given for each horse after each food ration distribution. Water was provided ad-libitum.

Horses of both groups underwent each morning an exercise of 30 min/day, during 3 successive days (Day −2, −1 and 0). Blood was sampled before and after exercise. Following the 3rd day exercise (Day 0) and its corresponding subsequent blood sampling, a plasmapheresis session was performed on the horses of the plasmapheresis group while horses of the control group were maintained in the same experimental environment but without being subjected to plasmapheresis. Then blood was sampled from each horse of both groups. Subsequent daily blood samples were taken at the same hour for the following 4 days (Day 1, 2, 3 and 4). All blood samples were obtained using jugular venipuncture. Sampling was realised in the morning between the two first food diets of the day.

In order to verify the effect of plasmapheresis, the physical exercise executed by horses have to comply the condition of inducing a significant raise of several hematobiochemical parameters and specifically providing moderate to high values of lactates. The well-known triangular exercise carried out in the trotter horses by Demonceau and Auvinet [2] have been first used. This exercise corresponds to succession of warm-up period (15 minutes) and 11 minutes of workloads exercise (steps) and then a recovery period of 10 minutes. Data of lactate concentrations obtained from blood samples taken 5–10 minutes after exercise test finished varied from 2.0 and 3.7 mmol/L (Fig. 1B). To obtain high values of lactates, the previous exercise test was modified by increasing the duration of the warm-up phase up to 30 min and steps were substituted by gallop. The new warm-up stage consisted on 5 min trot, 3 min of gallop, 5 min of trot, 3 min of gallop, 5 min trot, 3 min of gallop and finally 6 min of trot. The speeds of horses at trot was 240 m/min while it was 350 m/min for gallop. Data of blood lactate concentrations increased by 6 times (Fig. 1B) varying between 13.2 and 23.4 mmol/L. This new exercise was then used in the following experiment investigating the effect of plasmapheresis.

Plasmapheresis was performed by harvesting of 7L of plasma per horse which are replaced by 7L of NaCl 0.9%. The technique was performed by using a small-sized plasmapheresis machine commercialized by Hemofenix-France utilizing Trackpore technology (Dubna Moscow region-Russian Federation). The machine is equipped with Rosa® type membrane filters with 0.4μm pores. The system is totally automated operating.

Haematological parameters included the number of red blood cells (RBCs), hemoglobin concentration and hematocrit, the mean corpuscular volume (MCV), the mean corpuscular hemoglobin (MCH), the mean corpuscular hemoglobin concentration (MCHC), the number of platelets and also the number of white blood cells (WBCs) and the count of lymphocytes, monocytes, eosinophils and granulocytes. All these parameters were determined using a veterinary cell-counter automate, the Celltac VET MEK-6550 haematology (Nihon Kohden, Tomioka-Japan) and its reagents, Hemolynac·3 MEK-660I, Isotonac·4 MEK-641I, Cleanac MEK-520I (Celltac VET MEK-6550 J/K haematology, Nihon Kohden, Tomioka-Japan). Concerning the biochemical parameters, the concentrations of albumin, globulin, total protein, glucose, alkaline phosphatase (ALP), aspartate aminotransferase (AST), gamma glutamyl transferase (GGT), total bilirubin, lactate, creatinine kinase (CK), urea, creatinine, calcium, sodium and potassium were determined using the veterinary Skyla-VB1 automate analyzer (Lite-On Technology Corporation, Hsinchu-Taiwan). All the chemical reactions completed inside a circular and transparent plastic reagent disc (Equine Panel- Product code 900-150, skyla™ VB1 reagent disc, Hsinchu-Taiwan), containing different reaction cuvettes with specific assay reagent for each measured parameter. Concentrations quantified by photometric measurement of the absorbance changes arising from the chemical reactions in cuvettes’ disc are automatically given by the analyzer.

Lactate concentration was assayed using the portable analyzer Lactate-Pro™ 2. This test meter is based on a reaction of the blood lactate with a reagent in the Lactate-Pro™ 2 Test Strip (Busimedic®, S.L, San Sebastián-Spain). To validate and to ensure the accuracy of this technique, blood lactate concentrations measured using the Lactate-Pro™2 were compared to those measured by a reference analyzer, COBAS™ Gen.2 using the reagent COBAS INTEGRA Lactate Gen.2 (Roche®, Maylan-France). These analyses were conducted on specimens of six Athlete show jumping horses having performed the developed exercise, as previously described. Blood was sampled before and after the exercise and lactate concentration was immediately assayed by Lactate-Pro™2 and COBAS™ Gen.2 analyzers. Measurements from both instruments were tested for correlation.

**For further information concerning the experimental procedures** please see the research article [1] accompanying this data paper.
